# Mechanical Design Optimization of Prosthetic Hand’s Fingers: Novel Solutions towards Weight Reduction

**DOI:** 10.3390/ma15072456

**Published:** 2022-03-26

**Authors:** Federica Buccino, Alessandro Bunt, Alex Lazell, Laura Maria Vergani

**Affiliations:** 1 Department of Mechanical Engineering (DMEC), Politecnico di Milano, Via La Masa 1, 20156 Milano, Italy; federica.buccino@polimi.it (F.B.); alessandro.bunt@mail.polimi.it (A.B.); 2 Hy5, Bygning 18 Raufoss Industripark, 2830 Raufoss, Norway; al@hy5.no

**Keywords:** hand prostheses, lightweight design, finite element models, topology optimization, selective laser melting

## Abstract

From the mechanical function of grabbing objects to the emotional aspect of gesturing, the functionality of human hands is fundamental for both physical and social survival. Therefore, the loss of one or both hands represents a devastating issue, exacerbated by long rehabilitation times and psychological treatments. Prosthetic arms represent an effective solution to provide concrete functional and esthetical support. However, commercial hand prostheses still lack an optimal combination of light weight, durability, adequate cosmetic appearance, and affordability. Among these aspects, the priority for upper-limb prosthesis users is weight, a key parameter that influences both the portability and the functionality of the system. The purpose of this work is to optimize the design of the MyHand prosthesis, by redesigning both the proximal and distal finger and thumb in light of finding an optimal balance between weight reduction and adequate stiffness. Starting from elastic–plastic numerical models and experimental tests on obsolete components, analyzed under the worst loading condition, five different design solutions are suggested. An iterative topology optimization process locates the regions where material removal is permitted. From these results, 2 mm geometrical patterns on the top surface of the hand prosthesis appear as the most prominent, preventing object intrusion.

## 1. Introduction

**Figure 1:**
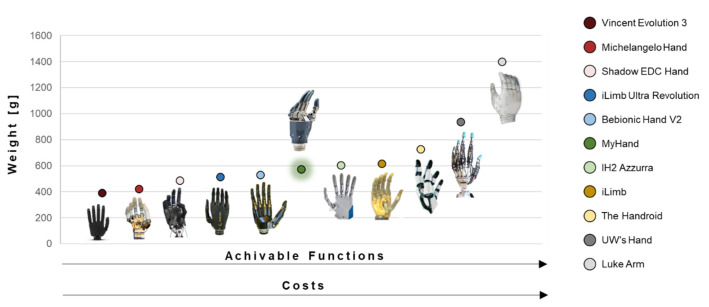
Comparison between weights of eleven anthropomorphic prosthetic hands. On the horizontal axis, the achievable functions and device costs increase from the left to the right. The MyHand prosthesis is highlighted with a green aura.

From the mechanical function of grabbing or holding objects to the emotional role of gesturing and communicating, the functionality of human hands is fundamental for both physical and social survival [1,2,3]. Therefore, the loss of one or both hands represents a devastating burden, exacerbated by long rehabilitation times and psychological treatments [4,5]. In this context, prosthetic arms represent an effective solution to provide concrete functional and esthetical support [6]. However, until recent times, the design of prosthetic limbs has progressed relatively slowly, limited by technological and material constraints [7]. Indeed, for centuries, hand prostheses remained passive devices, offering limited movement and almost non-existent control. The improvements in the mechanical design and the incorporation of hinges and pulleys into the prosthetic hand system led, in the 16th century, to the first examples of mechanical body-powered devices, such as metal hooks able to expand and narrow in accordance to user’s elbow bending. The dramatic increase in amputees after the two World Wars [8] gave a necessary boost to the implementation and testing of myoelectric prostheses, which amplify electromyographic (EMG) potentials from residual muscles at the amputation stump to power motorized parts [9,10]. Although converted into marketable devices, these models suffer from high weight, slow movement, weak pinch force, and delicate wire connections [11,12,13,14,15,16,17,18,19]. Remarkable advancements in increased control and bio-feedback [20] occurred in conjunction with the Paralympics [21], which gained attention both from media and high-tech companies, investing in this field [22,23,24,25,26,27]. Approaching an advanced trans-human integration between machine and body still represents an ongoing target goal in upper-limb prosthetic devices. Today’s arm and hand prostheses are commonly characterized by the following core components [28]: (a) the socket—this is the primary interface between the residual limb and the prosthetic hand, wrapping the end point of the residual limb and forming a suction/mechanical connection between the limb and the prosthesis; (b) the actual mechanical hand with a palm (containing the motor), up to four mechanical fingers, a mechanical thumb, and the respective motion transmission mechanisms (gears, wires, pulleys); (c) the electric motor(s) to open and close the fingers and thumb of the prosthesis (the most common actuator used in prosthetics today, excluding body-powered harnesses, is a direct current (DC) motor [28]); (d) the battery, ensuring electric power to the motor; (e) the control system, which exploits properly amplified EMG signals. These signals are recorded with surface electrodes, detecting electrical activity related to the patient’s remaining limb muscles, to control the motor. In this way, it is possible to interpret a voluntary intention of the user who controls the hand by appropriate muscle contraction [29]. However, commercial hand prostheses still lack an optimal combination of light weight, high functionality, durability, adequate cosmetic appearance, and affordability [28,30,31,32,33,34,35,36]. Among all these aspects, the deciding factor is the user perception and expectation, which covers all aspects of the device, from prosthesis control and appearance to daily use experience. One of the priorities for upper-limb prosthesis users is weight, a key parameter that influences both the portability and the functionality of the system [30,37,38]. In order to shed some light on the centrality of the weight constraint and the related impact in terms of hand motion functions, a detailed market analysis was carried out, considering the commercially available devices over the past ten years. Eleven anthropomorphic prosthetic hands were compared in terms of the weight of the entire system to be carried by the user (Figure 1) [28,38,39,40]. The weight is eventually inclusive of the battery, controller, force-sensing resistors used to simulate EMG electrodes, protective sleeves, wrist adapters, and quick-disconnect wrist unit. As expected, the weight has an effect on the overall size, number of joints, degrees of freedom, number of actuators, actuation method, joint coupling method, adaptive grip, grip force, range of motion, grasp type, motor, and finger flexion/extension speed. Jing et al. [40] point out that increasing the hand motion functions increases the weight, while reducing the weight leads to a simple mechanism that cannot achieve diversiform hand motions. All the hands that could achieve 13 types of motion weigh more than 600 g. Meanwhile, even though some of the lightweight hands weigh under 400 g, they cannot achieve more than six types of motion. This trade-off problem implies that combining dexterity and light weight is a great challenge for prosthesis producers.

Although the human hand has an average weight of 400 g, which is comparable with many devices shown in Figure 1, prosthetic hands with this weight have been reported by users as being too heavy, generating huge discomfort in daily activities [38]. However, this can depend on variables such as the age and gender of the user, as well as the length of the residual limb. It is therefore complex to establish universal mechanical and performance requirements [41,42], which are subjected to patients’ exact needs, including the nature and level of their amputation, as well as the level of activity and professional needs. Taking this into account, it is crucial that the manufacturer offers a selection of different sizes for the same prosthetic solution, in such a way that the user can choose the model that feels more comfortable and better matches her/his body characteristics. The increased customization of the provided solutions comes with an impactful drawback—high prices [43,44]—which, together with unfavorable reimbursement issues for amputees, compel customers to opt for lower-priced products that are less effective.

From this screening, the need for cost-effectiveness and lightness [45] emerges as a priority and an urgency to address amputees’ consumer requirements. Among the presented devices, MyHand, produced by Hy5 [46], is the focus of the present work. MyHand brings to the market the world’s first electro-hydraulically actuated prosthesis, optimizing the prosthesis weight and production costs by the exploitation of 3D printing techniques of the titanium fingers and plastic palm manifold. The power of the novel micro-hydraulic system lies in the possibility to grab even complex-shaped objects with an adaptive grip. The weight of the MyHand device is 575 g, in line with the available products on the market, but higher than the human hand weight, which is a common cause of discomfort during daily wearing.

**Figure 2:**
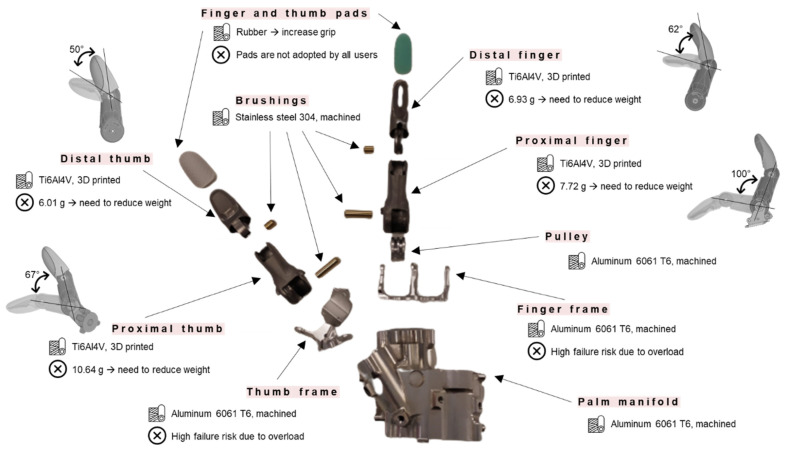
MyHand main components. Material and production process, together with rotations of proximal and distal components, and eventual criticalities experienced by users are highlighted.

An infographic of the main components of the prosthesis is reported in Figure 2, mentioning their characteristics in terms of material composition/realization technique/eventual allowed rotations and highlighting possible criticalities emerging from users’ experiences.

The user interface is given by two EMG sensors that allow the amputee to send the open/close signals thanks to the muscle’s contraction on the remaining part of the limb. The EMG sensors control a variable-speed motor that drives the hydraulic pumps of the system, which pull and release the cables connected to the index and middle fingers and thumb. The user can choose when to close/open the hand, and, unlike many of the prior state-of-the-art systems, the proximal and distal components in each finger will grip the object adaptively.

In accordance to Figure 2, the mechanical fingers represent a primary issue in terms of high weight (ranging from 6 to 10 g for each finger) and unoptimized design. In this context, this work aims at presenting a methodological approach to properly redesign the mechanical fingers of the device to balance weight reduction, sufficient mobility, strength, and cost minimization. The presented strategy exploits topology optimization on the proximal and distal finger and thumb phalanxes, which, thanks to the versatility of the manufacturing process, can be redesigned and 3D-printed in various shapes.

## 2. Materials and Methods

### 2.1. Design Targets

The set target is to reduce the overall mass of MyHand by at least 20 g acting on the fingers and thumb. Therefore, for each proximal and distal component, the target weight reduction is around 30%.

The constraints identified for the redesign phase are: (a) the external shape of the mechanical fingers and thumb must remain unaltered; (b) the mechanical fingers and thumb strength must not be significantly affected by the mass reduction; (c) the shape of the mechanical fingers and thumb must remain user-friendly, preventing human fingers or small objects from getting entangled; (d) the components must be designed considering the manufacturing process (selective laser melting (SLM) for the four components analyzed in this work, so wall thicknesses of at least 0.5 mm and holes with a minimum diameter of 2 mm).

On their product specifications, it is reported that MyHand prostheses can be statically loaded with 8 kg applied on the fingertip of the finger or thumb in full extension, without mechanical failures in the prosthetic hand. In obsolete MyHand prostheses, a high safety factor was considered to design proximal and distal components: for this reason, there is room for a new design that reduces the weight without significantly affecting the intended use of the structure.

To reach the mentioned overarching goals, a combined computational and experimental approach is adopted, towards the definition of an optimal weight-controlled redesign of prosthetic fingers.

### 2.2. Computational Methods

**Figure 3:**
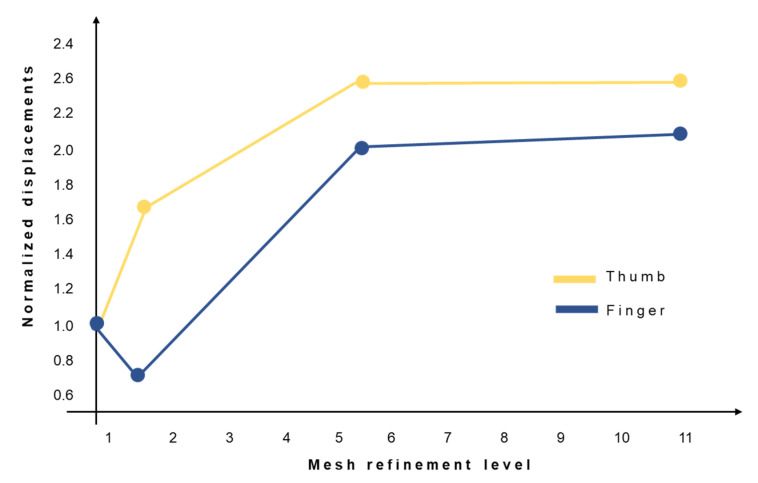
Mesh refinement process for both the obsolete finger (in blue) and thumb (in yellow).

Finite element (FE) models are implemented in Abaqus CAE (v. 2021, SimuliaTM, Dassault Systèmes^®^, Vélizy-Villacoublay, France) to analyze stress distributions in the proximal and distal components of MyHand and to evaluate candidate sites for design optimization. The performed analyses consider an elastic–plastic behavior (bilinear assumption) of the material, which is an aluminum alloy (AlSi10Mg) with an elastic modulus E = 70 GPa, yielding stress σ_y_ =190 MPa, ultimate tensile stress UTS = 300 MPa, and elongation at break ε_f_ = 2%. Two assemblies are considered: one for the finger (proximal and distal) and one for the thumb (proximal and distal), as depicted in Figure 2. A non-linear static general step is chosen, to properly consider the contact between parts; distal and proximal rotations are implemented with a kinematic coupling constraint, blocking all degrees of freedom except for the rotation around the distal or proximal axis, respectively. Boundary conditions are set on the proximal components: they simulate the proximal rotations and the hard-stops in contact with the respective frames. The hard-stops’ contact blocks the rotation when the finger or thumb is in the fully extended position and cannot rotate further. The load is applied on the distal components, to analyze the worst-case scenario where the load direction is applied on the fingertip and perpendicular to the open finger or thumb. It represents the real-life situation wherein the user falls and transmits all the weight to the open fingers and thumb. Three-dimensional models are considered with ten-node tetrahedral elements, adopted after performing mesh convergence analysis [47,48]. The mesh convergence is considered on four progressively increasing mesh densities for both the finger and the thumb. In Figure 3, the y-axis shows the normalized displacements with respect to the same quantity obtained with the coarsest mesh, while the x-axis reports the mesh refinement level, computed as the number of elements per assembly divided by the number of elements of the coarsest mesh assembly. Additionally, meshes are refined in the joints between proximal and distal components where intensifications of stresses are expected.

### 2.3. Topological Optimization

A condition-based topology optimization process is implemented in Abaqus CAE 2021 to locate possible material removal sites in the current design of the MyHand proximal and distal components.

**Figure 4:**
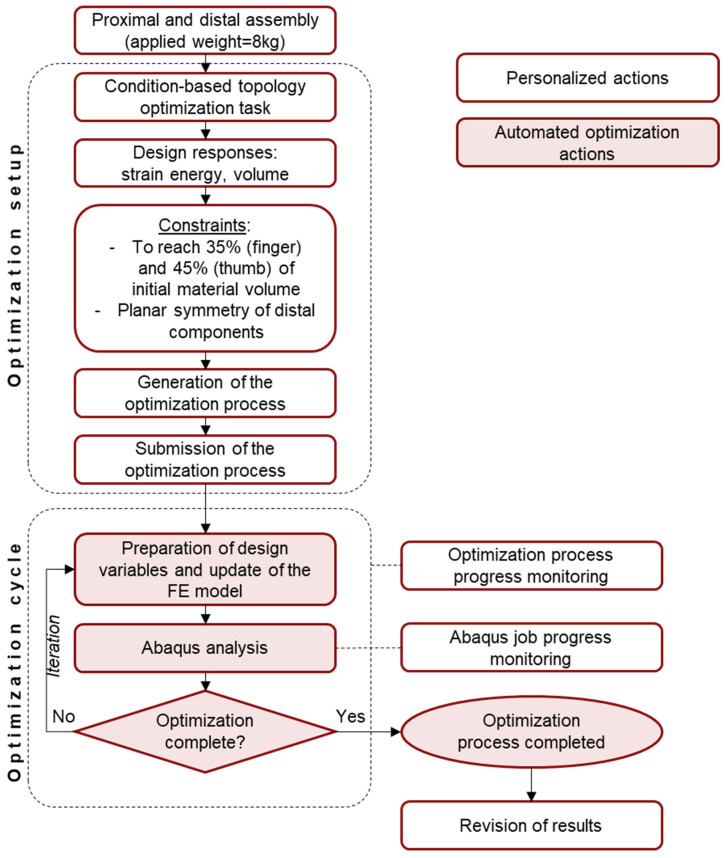
Optimization process adopted for the obsolete finger and thumb. Personalized actions are reported in white boxes. Automated optimization actions are reported in pink.

Topology optimization spatially improves the distribution of material within a defined domain (the assembly composed by the proximal and distal finger and thumb, respectively), by fulfilling given constraints, with the goal of maximizing the performance of the prosthetic hand. In this case, constraints specifically focus on material volume reduction, i.e., 35% for the finger and 45% for the thumb of the initial volume, without altering the external shape (volume of the element). Therefore, the optimization process determines a new material distribution by changing the relative density and the stiffness of the elements in the initial design [49]. The relative density of each element is defined as the ratio between the volume of material in the element and the volume of the element itself. The implemented process considers the strain energy and the stresses at the nodes to generate elements in the final design that are either void (their relative density is very close to zero) or solid (their relative density is equal to one). An iterative process of maximum 30 cycles is implemented in this work, considering the same mesh, contact, and boundary conditions detailed in Section 2.2. A distributed load, equivalent to 8 kg, is applied on the tip of the finger and thumb, as required in the product performance specifications. A schematic of the optimization process blocks is reported in Figure 4.

The results obtained by the topological optimization process require a further revision step in Autodesk Inventor Professional (v. 2021, Autodesk Inventor, San Rafael, CA, USA) to smoothen removed surfaces with critical sharp edges, which cause manufacturing issues in the SLM fabrication process. At the end of the process, five new design solutions are implemented and compared.

### 2.4. Experimental Campaign

**Figure 5:**
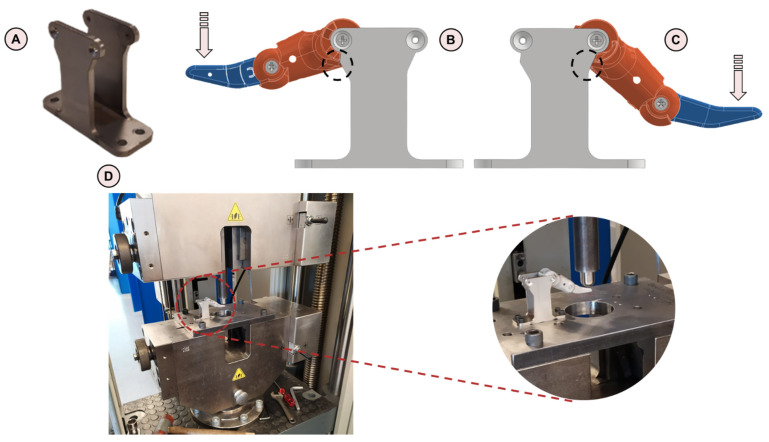
(**A**) Prosthetic finger and thumb support; (**B**) hard-stop inclination with respect to the horizontal plane of the thumb; (**C**) hard-stop inclination with respect to the horizontal plane of the finger; (**D**) Z250 Zwick Roell testing machine with a zoom on the tested specimen.

The experimental campaign requires the design of a prosthetic finger and thumb support (Figure 5A) to rigidly hold the devices in the open position with the distal component fingertip horizontal. In this way, the most critical loading condition is obtained when applying a vertical force on the tip of the component, corresponding to FE simulation. The support is realized in stainless steel S335, produced in two parts welded together. It can be noted that the supporting structure is not symmetric: this reflects two different hard-stop inclinations with respect to the horizontal plane of the thumb (Figure 5B) and the finger (Figure 5C). A Z250 Zwick Roell testing machine (ZwickRoell, Ulm, Germany) is adopted for compressive tests on the prosthetic devices (Figure 5D), which are performed under displacement control with a crosshead speed of 5 mm/min. A trigger of 20 N is used to start acquiring data when the finger or thumb is in the fully extended configuration, in the cases where it is not mounted in the completely open position. Three replicas of obsolete fingers and thumbs, respectively, are tested during the experimental campaign. These components are real devices already used by amputees. Additionally, 15 new design prototypes (3 for each of the 5 new design configurations), resulting from topological optimization and provisionally produced in PA12 with Selective Laser Sintering (SLS), are tested under the same loading conditions specified for obsolete components. The most prominent configuration will be later 3D-printed in Ti6Al4V.

## 3. Results

### 3.1. Analyses on Obsolete Components

**Figure 6:**
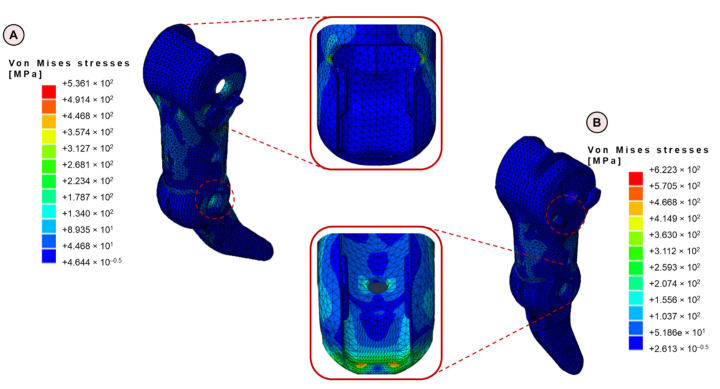
(**A**) Computational results in terms of Von Mises stresses for the finger; (**B**) computational results in terms of Von Mises stresses for the thumb. For both numerical models, a zoom on the regions subjected to higher stresses is reported.

Numerical and experimental analyses on obsolete components are the starting point for an adequate redesign that overcomes the highlighted weaknesses in the old design and manufacturing processes. Figure 6 shows the Von Mises stress maps on both the finger (Figure 6A) and the thumb (Figure 6B), as outputs of the numerical simulations, highlighting critical regions, where peak values of Von Mises stresses are reached.

It is possible to point out that the maximum values of Von Mises stresses are, for both finger and thumb, on the proximal components. For the finger, stress raisers are located at the thin edge near the base, at the hard-stop, and at the opening for the finger retaining strip, due to the presence of sharp edges. Concerning the thumb, the most stressed regions are the thin edge at the proximal thumb base, the hard-stop, and the thin edge near the distal rotation axis, due to smaller contact surfaces. Highest values of stresses are detected in the thumb, reaching peaks of 620 MPa, while lower values are detected in the finger (530 MPa).

**Figure 7:**
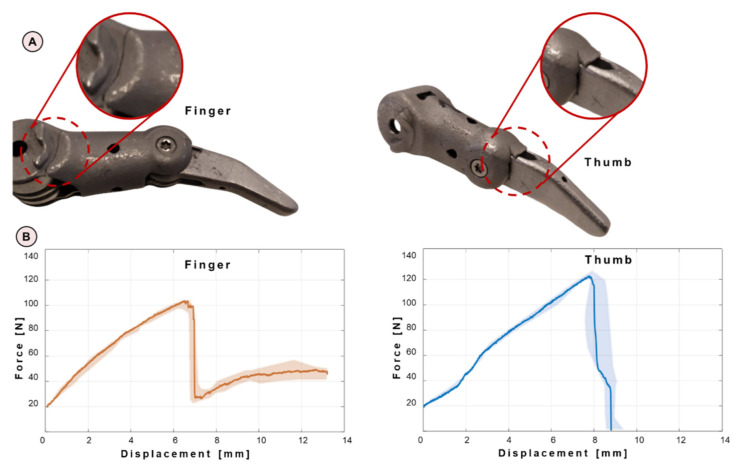
(**A**) Cracked regions from experimental tests on the finger (**left**) and thumb (**right**); (**B**) output in terms of force–applied displacement for both the fingers (**left**) and thumbs (**right**). The scatter of the experimental data is reported with pink (for the fingers, on the left) and light-blue bands (for the thumbs, on the right).

As regards mechanical tests on obsolete components, all failures occur in the proximal components and the cracks initiate in the same regions for the same assemblies. For the fingers, the crack starts propagating from the thin edge close to the base of the proximal finger (Figure 7A), while for the thumbs, two cracks initiate at the proximal–distal contact surface (Figure 7B). The output in terms of force–applied displacement is reported in Figure 7B for both the fingers and the thumb.

### 3.2. New Weight-Optimized Designs

**Figure 8:**
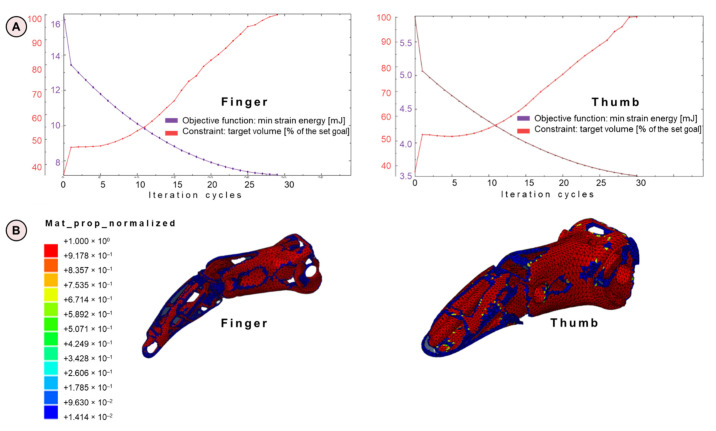
(**A**) Topology optimization graphs for the obsolete finger (left) and the obsolete thumb (right), reporting both the objective function and the constraint; (**B**) map of the relative density (mat_prop_normalized) for finger and thumb optimization. Blue regions are identified as possible removal sites, in contrast with red zones.

Finger and thumb components are optimized to maximize the weight reduction while keeping adequate mechanical properties in terms of the stiffness of the prosthetic structure. The topological optimization is characterized by an iterative process in which two functions are considered: on one hand, there is the objective function, which is a measure of the component mechanical characteristics, and on the other hand, there is the target volume to be reached (which is expressed as a percentage with respect to the set goal: 35% of the initial volume for the finger and 45% for the thumb). During the iterations (Figure 8A), the optimization process progressively removes material from the obsolete components while seeking for an optimized configuration; as a consequence, the overall stiffness decreases and the configuration approaches the target in terms of volume reduction (positive slope of the target volume curve). The solution generates voids in many regions of the MyHand components, as reported in Figure 8B, where the material property normalized is the relative density of each element.

**Figure 9:**
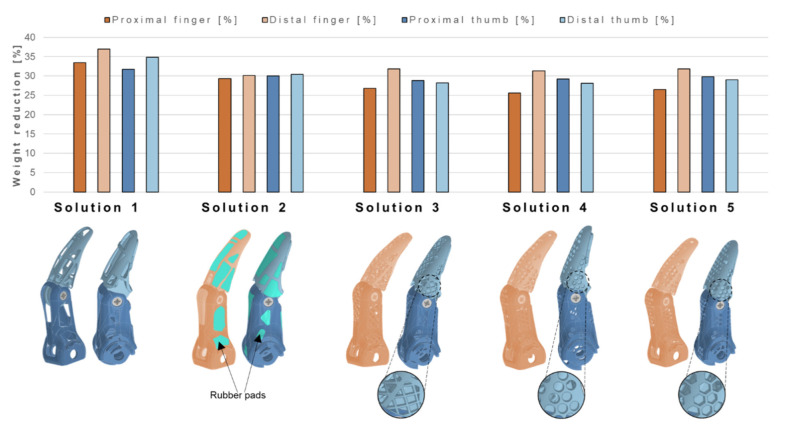
Weight reduction as a percentage of the obsolete component weight for proximal and distal finger and thumb of the novel-designed five solutions. On the bottom part of the image, a schematic of each solution is reported, with a zoom on their specific peculiarities (geometrical patterns).

From the identification of possible material removal sites, five different configurations are designed, always considering SLM constraints (Figure 9). The weight reduction percentage for both the proximal and distal finger and thumb with respect to the obsolete design is reported in the same figure. *Solution 1* shows the most extreme weight reduction, while solutions 2, 3, 4, and 5 are characterized by comparable values of weight decrease percentage. However, *Solution 1* shows the presence of large voids, where dirt and dust entering may affect the mechanical performance of the component. For this reason, *Solution 2* includes 16 rubber pads for the finger and 25 for the thumb, which can be glued on the component with epoxy glue, additionally improving the grip. *Solutions 3, 4*, and *5* tackle the challenge of making *Solution 1* more user-friendly and reducing void dimensions though grid patterns (*Solution 3*), circular voids (*Solution 4*), or hexagonal shapes (*Solution 5*).

Concerning experimental testing on new configurations provisionally realized in PA12, only one prototype shows the presence of a crack: in *Solution 5*, a fracture initiates on the proximal finger at the proximal–distal contact surface, but slows down its progression through the hexagonal pattern on the top surface of the component.

## 4. Discussion

In this work, the main issue of amputees, i.e., discomfort from high weight of the prosthetic device, is considered as a key aspect for the redesign of finger and thumb.

The starting point is the computational and experimental analyses of obsolete components, in order to identify critical regions, where material removal is prevented by the need for improved mechanical characteristics, and low stressed areas, where the redesign is highly recommended.

FE models are validated by experimental tests, reaching the same order of magnitude in terms of displacements (uFEM uEXP). The obsolete finger and thumb tested during the experimental campaign present a crack initiation in the regions with the highest Von Mises stresses identified by the computational models. More in depth, the use of plastic material behavior allows us to capture the stresses at the contact surface in the proximal thumb with increased accuracy in terms of stress distribution if compared to a linear elastic model. The experimental results show that the thumb is able to sustain higher forces with respect to the finger. This is mainly due to the two hard-stops that make the stress distribution in the thumb more symmetric and more uniform throughout the components. The finger, on the other hand, has a hard-stop on one side only and this reduces the strength of the proximal finger.

The 20% underestimation of displacements in the computational models with respect to experimental tests, despite not infringing FE model validity, brings to light three numerical approximations that are worth mentioning. Firstly, the surface supposed to be horizontal when the finger or thumb is connected to the support is actually inclined by around 5° due to the wear of the tested components (specifically on the distal–proximal contact surfaces). Secondly, the numerical displacement corresponds to the displacement of the midpoint of the surface where the equivalent pressure is applied, while the testing machine reports the vertical displacement of the crosshead. When applying the increasing displacement, the machine bends the finger or thumb and so the contact point is moving on the surface of the distal component, leading to over-dimensioned displacement in the last test phases. Thirdly, the numerical assemblies consider only the proximal and distal components, in order to reduce computational costs. The pulley, the cables, the internal and external springs, the strip to pull back open the fingers or thumb, the brake system of the obsolete finger, and the bushings are indeed not included in the numerical models. The focus is, in fact, on the proximal and distal components, but in the actual assembly, these parts influence the overall system.

Keeping a critical eye on the necessary simplifications performed in the numerical models, five novel solutions are proposed for the redesign of proximal and distal fingers and thumbs, presenting an encouraging weight reduction of 31 ± 6.9%. The main advantages and disadvantages of each solution, both in terms of mechanical/geometrical features and related costs, are presented in Table 1.

*Solution 1* is the most extreme in terms of weight reduction, with a maximum of almost 37% in the distal finger. However, the presence of large voids discourages the manufacturer from *Solution 1* further implementations. Indeed, it is observed that not all users adopt the protective silicone glove [50], which is designed to provide a natural appearance to the hand prosthesis and to protect interior mechanisms when exposed to water and dirt. For this reason, this first design lacks user-friendliness, because a small finger or a small object can become entangled, together with dust, affecting the opening and closing mechanisms. Additionally, it can be noted how the weight reduction is approximately 30% of the initial volume, less with respect to the optimization processes that removed 65% and 55% of the total volume. This difference is given by the SLM constraint considered while redesigning the components, which requires parts to have a maximum of 0.5-mm-thick walls, differently from the topological optimization process, where this limitation is not present. To seal the internal mechanism from foreign matter, a plastic film can be inserted with a thermoforming process to generate a barrier without significantly increasing the weight. However, the distal and proximal components of *Solution 1* would have to undergo this additional manufacturing procedure, where a film is placed on the external surface and heated to make it shrink over the component, conforming to its shape. However, the film, which comes with a relatively low cost, can become punctured, or wear may occur during everyday life, so a repair service should be offered to the user.

To overcome the drawbacks of *Solution 1*, *Solution 2* includes glued rubber pads, improving the grip of the prosthetic hand thanks to the higher friction coefficient of the pads with respect to the titanium surfaces (especially thanks to the pads on the bottom of the proximal components, which are often in contact with the grasped object). The low cost of rubber pads and their low density appear as a promising perspective; however, they imply greater assembly time and more complicate dismounting processes. Wear is also a problem, and it could lead to pad detachment, leading to the need for a repair service, as in *Solution 1*. For experimental tests, this solution suffers the greatest strength decrease with respect to the MyHand components due to the absence of the stiffening structures that fill the large voids.

*Solution 3, 4*, and *5* present the common idea of overcoming foreign matter intrusion with different patterns, which reduce the possibility of large objects becoming entangled inside the prosthetic device. *Solution 3*, with grid structures inclined by 45°, is chosen to maximize stiffness; indeed, diagonal bracing and ribbing are used in many lightweight design applications [51]. Both *Solutions 3* and *4* do not significantly reduce the strength as happens with *Solution 1* thanks to the stiffening grid configurations. From the experimental tests on the prototypes, *Solution 4* with the circular pattern results as the most prominent candidate in terms of weight reduction and adequate mechanical properties. *Solution 5*, which, to the naked eye, appears to be very similar to *Solution 4*, has a significant strength reduction. To properly exploit the lightweight honeycomb structures, the hexagonal holes must be made larger, with thicker connecting walls, so that they can be 3D-printed in a more precise way. However, this adjustment is in contrast with weight reduction and the need for a barrier to dirt and dust.

All the presented solutions succeed in significantly reducing the weight of the prosthetic device (25–30% finger weight reduction and approximately 4% entire prosthesis weight reduction); indeed, the topological optimization process results as a powerful tool to locate the regions for material removal, without significantly affecting the mechanical response of the component. Among the discussed options, the introduction of a geometric pattern shows some evident advantages in providing an optimal balance between weight and stiffness, simultaneously dealing with the issue of small object intrusion. Indeed, geometrical pattern dimensions could be adequately tuned in accordance to specific necessities. This will open new perspectives in the lightweight design of hand prostheses that could be easily customized without implying dramatic impacts on manufacturing process costs.

## 5. Conclusions

The main outcomes of the work could be summarized as follows:

- Elastic–plastic FE models on obsolete components are implemented to locate the regions characterized by high stresses where material removal is prevented.
- FE models are validated by experimental tests on old prosthetic devices. The discrepancies in displacement outputs, even though not influencing the validity of the model, are accurately evaluated and liked to two aspects: numerical over-simplification of the assemblies and bending of the prosthesis during the experimental test.
- Topology optimization is exploited to identify material removal sites, keeping a specific target volume (35% of the initial volume for the finger, 45% for the thumb).
- Five novel designs are presented and critically compared for both finger and thumb. *Solution 1* is the most extreme in terms of weight reduction, with a maximum of almost 37% in the distal finger. However, the presence of large voids that could lead to foreign matter intrusion discourages the manufacturer from *Solution 1* further implementations.
- From the experimental tests on the prototypes, *Solution 4* with the circular pattern results as the most interesting option in terms of weight reduction and adequate mechanical properties. The introduction of geometric patterns paves the way for novel lightweight designs applied to hand prostheses, whose features can be easily tuned and customized in accordance to users’ needs.

## Figures and Tables

**Table 1:** Advantages and disadvantages of each novel-designed solution, in terms of mechanical, geometrical features, and related costs.

	Potentialities	Critical Issues
**Solution** **1**	Maximum weight reduction (35% finger, 33% thumb) among presented solutions	Children’s fingers or small objects can get entangled Dirt and dust can penetrate the components Maximum strength reduction
**Solution 1** **+ Film**	Internal mechanisms are sealed from the outside	Extra thermoforming process to be added to production Puncture or wear of the film in everyday use Strength reduction analogous to *Solution 1*
**Solution** **2**	Internal mechanisms are sealed from the outside Improved grip thanks to rubber pads	Great number of pads to be glued on the components (assembly time increased, difficulties in disassembly) Pads can detach during everyday use Strength reduction analogous to *Solution 1*
**Solution** **3**	Improved strength with respect to *Solution 1*	Dirt and dust can get inside
**Solution** **4**	Lowest strength reduction	Dirt and dust can get inside
**Solution** **5**	Improved strength with respect to *Solution* 1 (limited increase)	Dirt and dust can get inside

## Data Availability

Data are contained within the article.
